# Utilizing polarization-selective mode shaping by chalcogenide thin film to enhance the performance of graphene-based integrated optical devices

**DOI:** 10.1038/s41598-019-48890-y

**Published:** 2019-08-27

**Authors:** Hamed Nikbakht, Hamid Latifi, Gholam-Mohammad Parsanasab, Majid Taghavi, Maryam Riyahi

**Affiliations:** 1 Laser and Plasma Research Institute, Shahid Beheshti University, Tehran, 1983969411 Iran; 2 Faculty of Physics, Shahid Beheshti University, Tehran, 1983963113 Iran; 3 Integrated Photonics Laboratory, Faculty of Electrical Engineering, Shahid Beheshti University, Tehran, 1983963113 Iran; 4 Faculty of Science, Shahid Chamran University of Ahvaz, Ahvaz, 6135743135 Iran

**Keywords:** Fibre optics and optical communications, Optical properties and devices

## Abstract

High refractive index (RI) thin films are capable of pulling waveguide mode profiles towards themselves. In this study, it is shown that by applying high RI coatings with specific thicknesses on the side of optical waveguides, significantly different mode profiles for orthogonal polarizations can be achieved. This phenomenon, that we call it polarization-selective mode shaping, can be extensively used in the enhancement of polarization-dependent integrated optical devices. As an illustrating application, a tri-layer structure consisting of poly(methyl methacrylate)/graphene/chalcogenide on a side-polished fiber is designed to realize an extremely high extinction ratio polarizer. This structure changes the mode profiles in a way that the attenuation of TE mode is maximized, while the power carried by the TM mode remains relatively constant. Simulations and experimental characterizations confirm that polarization-selective mode shaping coordinates four loss mechanisms to maximize the extinction ratio and minimize the insertion loss of the polarizer. The fabricated polarizer is examined in the O, C, and L telecommunication frequency bands. This configuration achieves the high extinction ratio of 51.3 dB and its maximum insertion loss in the tested wavelengths is 1.79 dB. The proposed polarizer has been compared with other state-of-the-art polarizers in the conclusion section which shows its superiority.

## Introduction

The interaction of light in waveguides with the surrounding media depends mainly on the penetration of the evanescent field in that media, which depends on the mode profile. There has been numerous researches on modifying waveguide structures to increase or decrease this interaction^1–3^. Among these structures, plasmonic coatings are capable of inducing significant difference between orthogonal polarizations. However, these structures usually work in a narrow frequency band and possess small penetration depth^4,5^. As an alternative, dielectric coatings can change the mode profiles into different shapes for orthogonal polarizations, and can overcome both of these limitations. However, they have not been extensively used for polarization-selective mode shaping (PSMS) and their ultimate functionality has not been revealed yet. The development of such dielectric structures can lead to various functionalities in integrated optical devices that do not suffer from the ohmic loss of plasmonic structures.

Chalcogenides, amorphous compounds containing sulfur, selenium, or tellurium, show interesting optical properties such as transparency in near- and mid-infrared, third-order nonlinearity, and waveguiding^6^. These properties has made chalcogenide a serious candidate for future integrated optical devices. In addition, chalcogenides can be easily coated on various substrates with different methods and low-cost equipments^7–9^. Considering these properties along with its high refractive index (RI), chalcogenide is an excellent choice to be used as the coating which induces PSMS.

Controlling the polarization of light in integrated optical devices is an outstanding application that can illustrate the potential of PSMS. A usual component in integrated optics that can benefit from PSMS is light polarizer. This component plays a crucial role in optical systems, especially in communications, sensors, and the interaction of light with anisotropic media. Along with the development of optical fiber technology and integrated optics, the demand for in-line polarizers is increasing. Utilizing these polarizers in optical systems results in more compact, alignment-free, and fully integrated configurations. In recent years, modified photonic crystal fibers^10–12^, plasmonic structures^13–15^, tilted fiber Bragg gratings^16,17^, and microfiber couplers^18^ have been used to realize in-line polarizers. However, they are still far away from the specifications of state-of-the-art free-space polarizers.

Graphene, the two dimensional (2D) allotrope of carbon, has been the subject of numerous researches since its isolation as a single layer in 2004^19^. Soon after its introduction as a stable material, theoretical investigations have revealed its promising properties and a lot of devices have been made to harness its capabilities^20^. Graphene have been used for light modulation^21,22^, ultra-short pulse generation^23,24^, wavelength conversion^25,26^, and sensing^27–31^. Due to the 2D structure of graphene, this material exhibits some orientation-sensitive properties. One of these properties is the polarization-dependent absorption of light^30,32^, which makes graphene an ideal material to examine PSMS on it. This property was used by Bao *et al*.^33^ to realize a broadband fiber polarizer. After their work, a lot of papers were published reporting the utilization of graphene or graphene oxide in waveguide- or fiber-based polarizers^34–41^. To enhance the extinction ratio (ER) in some of these polarizers, graphene was used in conjunction with metal^38^ or dielectric coatings^34,36,39^. However, these efforts did not satisfy the demand for in-line polarizers, mostly due to their high insertion loss (IL) and low ER.

In the current study, the capability of high RI thin films to induce contrast in orthogonal mode profiles is investigated numerically. To confirm these results experimentally and illustrate the potential applications of PSMS, a graphene-based in-line fiber polarizer (GILFP) is designed, fabricated, and characterized. This polarizer benefits from poly(methyl methacrylate) (PMMA)/graphene/As_2_S_3_ (chalcogenide) tri-layer coating on the top of a side-polished fiber (SPF). This structure reduces the power carried by the TE mode more than that by the TM mode through four loss mechanisms. First of all, at low Fermi energy levels (<0.4 eV), the absorption of TE-polarization is intrinsically higher than TM-polarization in graphene^22,32,41^. The PMMA and As_2_S_3_ coatings also create PSMS so that the interaction of the TE mode with graphene is increased. In addition, the mode coupling loss of the TE-polarized light is higher than that of the TM-polarized light in the transitions at the beginning and end of the polished region. Finally, the TE mode experiences more scattering loss due to its propagation near the polished surface. All these four loss mechanisms reduce the transmitted power through the TE mode while imposing a minor reduction on the power carried by the TM mode. In the absence of PSMS, the last three mechanisms would not necessarily result in higher loss for TE mode. But, this coating which reshapes TE mode, makes sure that the loss induced by these mechanisms is coordinated with the intrinsic loss induced by graphene. Therefore, in the output of the fiber, the power of the TM polarization is much more than that of the TE polarization and the device works as a TM-pass polarizer. This device polarizes light with high ER and low IL in a wide range of frequencies in the telecommunication bands. Owing to the uniform optical response of graphene in a broad spectral range, this method can be used to realize polarizers for other wavelengths with appropriate fibers and coatings.

In what follows, in the first step, the variations of different polarizations in the response to changes in the coating structure are presented. Then, the influence of these variations on the attenuation of modes in the presence of graphene is studied. Afterwards, the coupling losses at the transition regions are calculated. In the next step, the experimental characterization of the fabricated polarizer including scanning electron microscopy (SEM), Raman spectroscopy, and optical performance investigations is presented. Optical performance of the SPF with different coating configurations is also investigated to validate the proposed loss theories. Finally, a comparison between the state of the art optical fiber polarizer and the polarizer fabricated with this method is presented. The simulation, fabrication, and characterization procedures are described in detail in the methods section.

## Device Structure

**Figure 1:**
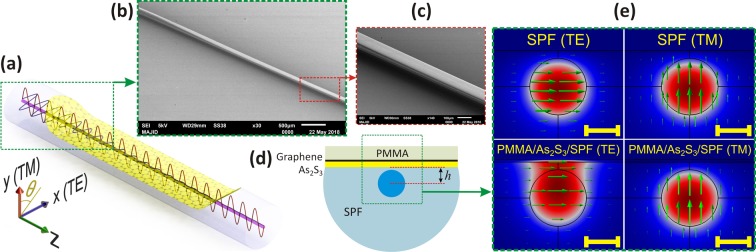
(**a**) 3D schematics of the GILFP. The propagations of the TE and the TM modes are illustrated as sine waves. (**b**) SEM micrograph of the SPF at one of the transition regions, (**c**) SEM micrograph of the SPF at the completely polished section, (**d**) schematic cross section of the GILFP structure, (**e**) simulated TE & TM mode profiles of the SPF with and without PMMA/graphene/As_2_S_3_ coating. The arrows illustrate the magnitude and direction of the electric field (The scale bars are equal to 5.0 *μm*).

High RI thin films draw the mode profiles toward themselves resulting a change in mode profiles. However, this mode reshaping occurs for both TE and TM modes, the coatings can be engineered to achieve different mode shapes for different polarizations. This property of high index thin films is utilized in this work to maximize the interaction of the TE mode with graphene, while the interaction of the TM mode remains almost unchanged. To implement this phenomenon in a fiber polarizer, different coating structures were simulated and finally a tri-layer structure containing PMMA/graphene/As_2_S_3_ on a SPF revealed the best results (See Supplementary Information for details). To fabricate this device, an As_2_S_3_ layer is coated on the polished surface of an SPF. Then, a graphene layer is transferred to the top of this layer and a PMMA overlay covers this structure (Fig. 1a–d). TE and TM modes of a bare SPF and an SPF with PMMA/As_2_S_3_ coating are shown in Fig. 1e. As can be observed in this figure, the TM mode has a minor sensitivity to the coatings on the SPF, while the TE mode is significantly affected by the coatings.

This tri-layer structure is designed as was mentioned above to maximize and minimize the ER and the IL of the polarizer, respectively, and to overcome the fabrication limitations as well. Simulation results which will be presented later in the text show that the As_2_S_3_ coating is necessary for maximizing the loss of the TE mode. As the absorbing element, graphene must be placed on top of As_2_S_3_. Otherwise, it will be degraded in the sputtering process used for coating the As_2_S_3_ layer^42^. Also, without the PMMA layer, a thicker As_2_S_3_ is required to be coated on the SPF. With this thick layer, two problems will arise. The first one is the high sensitivity of the mode profile to the thickness of the As_2_S_3_ layer. In this condition, a minor error in the As_2_S_3_ layer thickness will result in a large deviation from the optimized conditions. Also, with a thick As_2_S_3_ layer, the mode profiles is highly sensitive to the distance between the polished surface and the fiber center (polished depth, h in Fig. 1c) of the SPF, and a minor error in determination of this value will lead to deviation from the optimal conditions, too. The second problem is the exposure of graphene layer to the contamination in the absence of the PMMA layer. The PMMA overlay protects the graphene layer from contamination, ensuring the long-term functionality of the polarizer. Contamination both reduces the electrical conductivity of graphene and increases light scattering in the fiber. Therefore, with the proposed tri-layer structure, graphene does not degrade during the sputtering process and the interaction of the TE mode with the graphene layer can be maximized. Further, the PMMA layer thickness can be finely adjusted throughout the experiment which obviates the need for an accurate polished depth measurement. Also, the lifetime of the device is extended.

The following reasons are also considered for the selection of coating materials. As_2_S_3_ is used as a high index material (*n* = 2.43) considering its transparency at telecommunication wavelengths. Besides, the low difference in the electronegativity of As and S results in a low polarization in the As_2_S_3_ structure. Therefore, it has a minor impact on the graphene conductivity, and as a consequence, the conductivity of graphene on an As_2_S_3_ substrate is higher than that on an SiO2 substrate^43^. PMMA is used as the cover layer since it is commonly used for transferring graphene and its thin thicknesses can be simply achieved by spin coating.

## Simulation Results and Discussions

Three sets of simulations were conducted to verify the proposed reasons for TE mode attenuation. In the first set, the effects of different coating thicknesses on the mode profiles and on the interaction of light with graphene were studied. This simulation also confirms the concept of PSMS and illustrates the role of chalcogenide as a high RI material on this phenomenon. In the second simulation set, the attenuation of the modes for different coating thicknesses was numerically calculated. These simulations were performed by using the three dimensional (3D) beam envelope study of COMSOL MultiPhysics 5.2 software. In the third set, the losses at the transition regions were calculated using 2D finite difference time domain (2D-FDTD) solution of the Lumerical software. The parameters used in these simulations are presented in Table 1. In addition to these simulations, an analytical study of 2D counterpart of this waveguide is presented in the Supplementary Information, which proves that formation of different mode profiles for different polarizations is a direct consequence of Maxwell’s equations.

**Table 1:** Parameters used for the simulation.

Parameter	Value
length of polished section (mm)	17
length of transition region (mm)	1.73
polished depth (*μm*)	5.2
core RI	1.4711
cladding RI	1.4660
core diameter (*μm*)	8.2

### Mode shaping and propagation loss simulation

**Figure 2:**
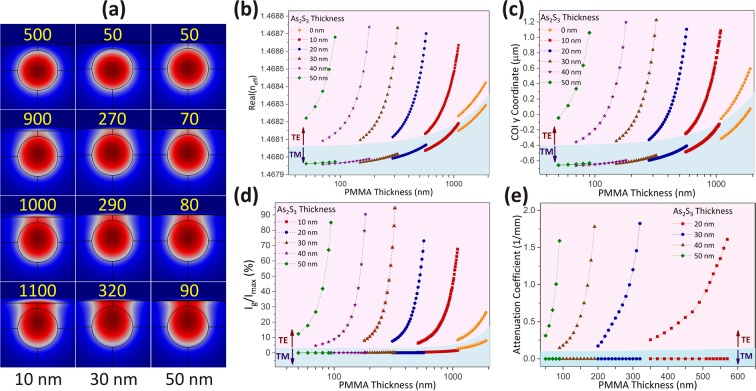
(**a**) TE Mode profiles for different thicknesses of As_2_S_3_ and PMMA. The value below each column shows the thickness of As_2_S_3_ and the value inside each image represents the PMMA layer thickness. All of these values are expressed in nanometers unit and the scale bare is the same for all mode profiles and is equal to 5.0 *μm*. (**b**) Effective refractive index, (**c**) y-coordinate of the COI, (**d**) the ratio of intensity on graphene layer to the maximum intensity of the mode, (**e**) attenuation coefficients for TE & TM modes for different As_2_S_3_ thicknesses versus PMMA layer thickness. The points in pink backgrounds belong to TE modes and the points in blue background represent TM modes.

Mode profiles for various thicknesses of the coated layers are illustrated in Fig. 2a. The effective RI is plotted for the various thicknesses of As_2_S_3_ and PMMA in Fig. 2b. It can be inferred from the plot that the effective index of the TE mode increases as As_2_S_3_ and PMMA thicknesses increase. However, the effective index increase for the TM mode is much slower. Due to the higher RI of As_2_S_3_, the impact of its thickness on the effective RI is significantly more than that of the PMMA.

To further investigate the effect of these coatings and optimize the thicknesses of the layers, two parameters are defined and calculated through the simulation. The first parameter is the y-coordinate of the center of intensity (COI) for each mode which is a measure of mode position. It can be calculated as:

1 $${y}_{COI}=\frac{\int \,y\,I(x,y)\,dx\,dy}{\int \,I(x,y)\,dx\,dy}$$

where, *I*(*x*, *y*) is the mode intensity in a plane perpendicular to the direction of propagation (z), and the integration is performed over this plane in the entire simulation domain. The second parameter is the ratio of mode intensity on the graphene layer (*I*_*g*_) to its maximum (*I*_*max*_) on a plane perpendicular to the propagation direction.

Figure 2c,d shows the behavior of these parameters relative to the PMMA layer thickness for various As_2_S_3_ thicknesses. It is evident that for the TE mode, COI moves upward as the thicknesses of As_2_S_3_ and PMMA increase. Also, *I*_*g*_/*I*_*max*_ approaches unity as these thicknesses increase. However, for TM mode, the change of these parameters is virtually zero. The attenuation coefficients due to the interaction of different polarizations with graphene in the SPF were also calculated through COMSOL simulations (Fig. 2e). These four parameters have been plotted for thicknesses in which the SPF is single-mode (Fig. 2b–e). The last point in each curve represents the thicknesses in which the structure becomes multi-mode.

According to these results, the maximum ER can be achieved for 30–50 nm thickness of the As_2_S_3_ layer. However, the sensitivity of these parameters to the PMMA layer thickness for a 40 nm or 50 nm As_2_S_3_ layer is too high which means that a very high accuracy is required in the PMMA layer thickness to achieve the maximum ER. Therefore, an As_2_S_3_ layer with a 30 nm thickness was used in this experiment.

### Coupling loss

**Figure 3:**
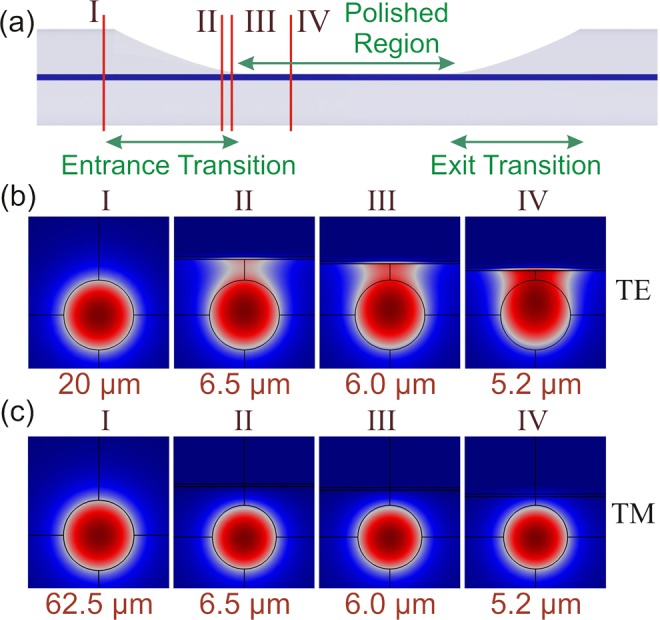
(**a**) Schematic side view of the GILFP, (**b**) TE mode profiles at different points in the transition region, (**c**) TM mode profiles at different points in the transition region. The letter above each profile designates the plane to which the mode profile belongs, and the values below the graphs show the polished depth of the structure at each plane.

As was briefly discussed above, there is a coupling loss for the TE mode in the transition regions which enhances the ER of the polarizer. TE and TM mode profiles at four different points in the “entrance transition” region are illustrated in Fig. 3. As TE mode propagates in the entrance transition region (Fig. 3a), it undergoes couplings from a symmetric mode (plane I in Fig. 3a) to a D-shaped one (plane IV in Fig. 3a). On the contrary, the TM mode profile does not change significantly (Fig. 3c). A reverse mode deformation is experienced by these modes as light propagates in the “exit transition”. Due to the higher deformation of the TE mode, a higher loss for it is expected compared to the TM mode.

The propagation of light in the transition regions was studied in more detail by using the 2D-FDTD simulation. The coupling efficiencies for TE and TM modes at the entrance and exit transition regions are presented in Table 2. Also, the video of light propagation in the transition regions for the TE and the TM modes is available in the Supplementary Information.

**Table 2:** Coupling efficiencies of TE and TM modes at the transition regions.

	TE	TM
coupling efficiency in entrance transition (%)	59.7	87.2
coupling efficiency in exit transition (%)	39.5	92.2
total coupling efficiency (%)	23.6	80.4
total coupling loss (dB)	6.27	0.95

## Experimental Results and Discussions

**Figure 4:**
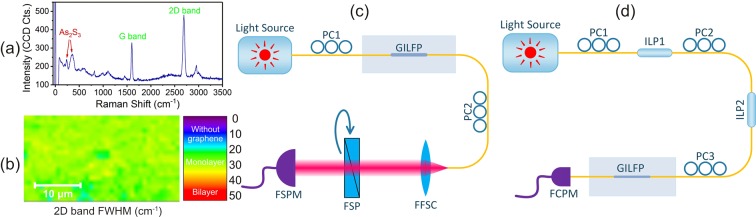
(**a**) Raman spectrum of the tri-layer structure. Graphene and As_2_S_3_ bands are labeled. Other peaks belong to PMMA, (**b**) surface mapping of the 2D peak FWHM of Raman spectrum over a 36 *μm* × 19 *μm* area of the coatings on the SPF polished section which shows that the graphene layer is uniform and monolayer (FWHM < 40 cm^−1^)^44^, (**c**) experimental setup for the measurement of transmitted power at different polarization angles, (**d**) experimental setup for accurate ER measurement. (PC: polarization controller, ILP: in-line polarizer, FFSC: fiber to free space coupler, FSP: free space polarizer, FSPM: free-space power meter, FCPM: fiber coupled power meter).

The Raman spectrum of the tri-layer structure is shown in Fig. 4a. This spectrum confirms the presence of graphene, PMMA, and As_2_S_3_. The full width at half maximum (FWHM) of the 2D Raman band was monitored in a scan over the polished surface of the fiber which confirmed the presence of a single-layer graphene on the fiber (Fig. 4b)^44^.

**Figure 5:**
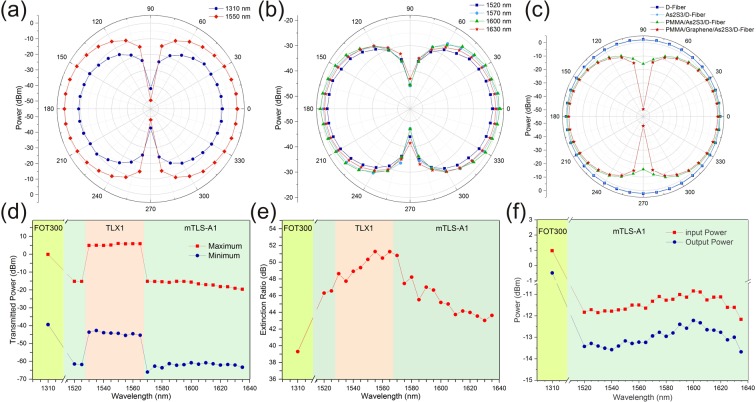
(**a**,**b**) Transmitted power from the GILFP at different polarization angles for different wavelengths, (**c**) transmitted power from the SPF at different polarization angles for different coatings, (**d**) the maximum and minimum powers achieved by adjusting the incident polarization on the GILFP. The jumps in this graph are due to using sources with different output powers. (**e**) ER of the GILFP vs. wavelength, (**f**) input and maximum output power of the GILFP for different wavelengths whose difference is equal to the IL of the device. The background colors in sub-figures (**d–f**) represent using different sources for different spectral regions.

Two different configurations were used to characterize the optical performance of the GILFP (Fig. 4c,d) (see methods section for details). The first one (Fig. 4c) measures the transmitted power from the GILFP projected into various linear polarizations. Figure 5a,b shows the results of this experiment for some selected wavelengths. As was expected, the power behaves as a *cos*(*θ*)^2^ function versus angle for the tested wavelengths.

The first configuration was also used to investigate the effect of different coatings on the response of the polarizer. For this purpose SPFs with different coatings were placed in this setup and the transmitted power in various linear polarizations was measured (Fig. 5c). The results show that the bare SPF and As_2_S_3_ coated SPF have a very low influence on the polarization. However, PMMA/As_2_S_3_ coated SPF shows about 17.4 dB power difference for perpendicular polarization angles which confirms the TE mode coupling loss in the transition regions and scattering loss in the polished region. As was expected, the highest ER is achieved when graphene is between PMMA and As_2_S_3_ layers.

The second setup (Fig. 4d) was used to accurately measure ER of the polarizer. This configuration measures the transmitted power from the GILFP when the incident polarization is parallel and perpendicular to the transmission axis of the GILFP. When the incident polarization is parallel to the transmission axis the transmitted power is maximum, and vice versa. Figure 5d presents the maximum and minimum of the transmitted power. The jumps observed in this graph are due to using different sources with different output powers. Since, ER is defined as the difference between the maximum and the minimum transmitted power from a polarizer in dBm units, using different sources has no influence on the measured ER (Fig. 5e), and this value is independent of the source used. These results show the maximum ER of 51.3 dB at the wavelength of 1555 nm. The ER at the C-band and L-band is higher than 47.7 dB and 43.6 dB, respectively. Also, the ER at 1310 nm is 39.3 dB. The comparison of these results with state of the art fiber polarizers is presented in Table 3.

**Table 3:** Comparison of the fabricated polarizer with the most significant and recent in-line polarizers.

reference	operating	Max.	
	wavelengths (nm)	ER (dB)	IL (dB)
Zhou *et al*.^17^	1500–1610	33	—
Xuan *et al*.^11^	1500–1620	30	7
Konoplev *et al*.^14^	980	41	—
Lin *et al*.^43^	1550	20.5	2
Bao *et al*.^33^	480–1650	27	—
Qian *et al*.^12^	1480–1600	18	1.5
Kim *et al*.^40^	1310	20	10
Yan *et al*.^16^	1525–1608	46	—
Dong *et al*.^13^	400–1600	27	—
Lim *et al*.^35^	1530–1630	40	5
Romagnoli *et al*.^10^	1510–1600	36	8
Pei *et al*.^34^	1530–1610	27.3	9
Zhang *et al*.^18^	1540–1570	26	—
Ahmad *et al*.^37^	1550–1600	35.7	6
Li *et al*.^38^	1525–1575	27	5
Zhang *et al*.^36^	1425–1600	37.5	1
Chu *et al*.^39^	1560–1630	44	10
Yahyaei *et al*.^15^	1550	38	—
current work	1520–1635	51.3	1.79

The IL of the polarizer is defined as the ratio of the transmitted TM mode power to the incident TM mode power of the GILFP. The IL measurements show that the IL of the fabricated polarizer is lower than 1.79 dB over the tested wavelengths (Fig. 5f).

## Conclusions

In summary, we have proposed a dielectric coating which is capable of creating different mode profiles for TE and TM modes. This difference in mode profiles is so that the interaction of different polarizations with the surrounding media is significantly different for orthogonal polarizations. This method is examined on an in-line polarizer which benefit the most from the difference in mode profiles. This polarizer was created by a tri-layer coating on an SPF. The effect of these coatings on the increase in the TE mode loss due to the enhancement of light-graphene interaction, and mode coupling was described and validated experimentally. The fabricated polarizer achieves the maximum ER of 51.3 dB which, to the best of our knowledge, is the highest reported value for fiber polarizers. Also, the insertion loss is the least value among high ER fiber polarizers. This polarizer covers the entire C and L communication bands with the ER of >47.7 *dB* and >43.5 *dB*, respectively. This polarizer is also capable of polarizing light at 1310 nm wavelength with the ER of 39.3 dB. A comparison of the fabricated polarizer with other recent in-line polarizers is presented in Table 3. The fabrication of this polarizer is not complicated and is fully compatible with ordinary graphene transfer mechanisms. Furthermore, by changing the utilized fiber and adjusting coating thicknesses, the polarizer can be designed for other wavelengths in the visible and near-infrared regions.

## Methods

### Mode shaping and propagation loss simulation

The parameters used in the simulation of the GILFP are presented in Table 1. The lengths of the polished region and the transition regions were acquired through the SEM analysis. The polished depth of the SPF was also estimated through liquid drop experiment (see Supplementary Information for details). The simulations were performed for a range of coating thicknesses in which the structure remained single-mode. Also, a linear slope in the transition region was assumed.

The graphene layer was modeled as a conductive boundary supporting the surface current density of *J* = *σE*. This surface current density changes the boundary condition for the tangential magnetic field at the As_2_S_3_ and PMMA boundary to *H*_*ta*_ − *H*_*tp*_ = *σE*_*t*_, where, *H*_*ta*_ and *H*_*tp*_ are the tangential magnetic fields at the boundary just inside the As_2_S_3_ and PMMA layers, respectively, and *E*_*t*_ is the tangential electric field at the boundary. Graphene’s conductivity (*σ*) is calculated by the Kubo formula^45^

2 $$\sigma (\omega )=\frac{i{e}^{2}\mu }{\pi {\hslash }^{2}(\omega +i{\tau }^{-1})}+\frac{i{e}^{2}}{4\pi \hslash }\,\mathrm{ln}(\frac{2|\mu |-(\omega +i{\tau }^{-1})\hslash }{2|\mu |+(\omega +i{\tau }^{-1})\hslash })$$

and implemented in the simulations. In this formula, *e* is the charge of an electron, *ω* is the angular frequency of light, ℏ is the reduced Planck’s constant, *τ* is the electron relaxation time and *μ* is the chemical potential which is assumed to be 0.08 *eV* for graphene on the As_2_S_3_ substrate. The chemical potential is lower than the case when graphene is on an SiO_2_ substrate due to the lower doping introduced by As_2_S_3_ to the graphene.

The simulations were conducted by using 3D beam envelope study of wave optics module of COMSOL Multiphysics 5.2. To calculate the attenuation of the reshaped modes in the polished region, 1 mm length of the tri-layer structure on the SPF was simulated and the attenuation for the total length was calculated from the attenuation in this region. Assuming that attenuation per unit length is constant (*P* = *P*_0_*e*^−*αz*^), the total attenuation can be calculated as:

3 $${T}_{tot}={T}_{0}^{{l}_{t}/{l}_{0}}$$

where, *l*_*t*_, *l*_0_, and *T*_0_ are the total polished length, simulated length, and the transmission coefficient of the simulated length, respectively. Due to its long computation time, this simulation was performed for fewer points than mode simulations.

### Coupling loss simulation

2D-FDTD simulation was used to investigate the coupling losses at the entrance and exit transitions of the GILFP. This simulation was performed in FDTD solution of Lumerical software. Uniform coatings with the thicknesses of 320 nm and 30 nm were considered for PMMA and As_2_S_3_ layers on the entire polished surface, respectively. However, due to the simulation of the structure in 2D, different core width and polished depth are required to achieve a single-mode propagation and a similar mode deformation. The field profile at the end of the transition regions was extracted and the square value of its overlap integral with the mode field was considered as the coupling efficiency. The product of the coupling efficiency at the entrance and exit transition regions is equal to the overall coupling efficiency.

### GILFP fabrication

Side polished fibers (SPFs) were bought from Phoenix photonics with a nominal distance between the polished surface and the fiber center (polished depth) of 5 ± 1 *μm*. At the first step, the SPFs were cleaned with acetone and 2-propanol. Then, they were rinsed with deionized (DI) water and blow-dried with nitrogen. A 30 nm thick *As*_2_*S*_3_ layer was coated on the polished surface of the SPF by radio-frequency sputtering. Single layer chemical vapor deposited (CVD) graphene on copper with 60 nm PMMA overlay (Purchased from Graphena) was cut to a 1 × 3 *cm* piece. Since graphene layers are coated on both sides of the copper foil, firstly, the bottom graphene layer was dissolved in nitric acid. Then, the copper foil was etched with 10% w/w iron(III) nitrate solution in DI for 35 min. Afterwards, it was transferred to another iron(III) nitrate solution with 1% concentration for 20 min, where the copper foil was fully dissolved. Then, a microscope slide which the SPF was fixed on it was placed in the middle of a crystallizing dish, and it was leveled as much as possible. The container was filled with DI water so that the water level was a few centimeters higher than the SPF. After that, the PMMA/graphene layer was place on the top of water in this container. Afterwards, the water was drained gently by opening a valve at the bottom of the container. During the water depletion, the PMMA/graphene layer was gently guided to the top of the SPF. If the slide is properly leveled, when the water surface reaches the slide, the PMMA/graphene layer remains at the SPF on the microscope slide and the water leaves the slide. After transferring the graphene to the SPF, the slide was placed in an oven at 60 C for three hours to vaporize the remaining water.

### Optical characterization

#### Measurement of transmitted power at different polarizations

To investigate the performance of the polarizer, two different optical test configurations were used. In the first one (Fig. 4c), a polarization controller (PC) was placed after the light source to adjust incident polarization to the GILFP. The transmitted light through the GILFP was coupled to free space after passing through another PC and a fiber to free-space coupler (FFSC) (Thorlabs, CFC-5X-C). The output beam was passed through a free-space polarizer (FSP) (Thorlabs Glan-Laser polarizer, GL-10) and its power was measured by a free space power meter (FSPM) (Thorlabs, S122C). This setup was used to measure the transmitted power versus different orientation angles of the FSP in a wide range of wavelengths. To perform the test for 1310 nm and 1530–1565 nm EXFO FOT300 and Thorlabs TLX1 were used as the light source, respectively. Also, JDSU mTLS-A1 was used for light source in 1520–1525 nm and 1570–1635 nm wavelength ranges. The output light of these sources is partially polarized. Therefore, to characterize the GILFP independent of the source polarization, PC1 was adjusted to maximize the transmitted power from the GILFP, then it was adjusted again to reduce the power by 50%. In this condition, the amplitudes of the TE and the TM modes of the incident light in the GILFP are approximately equal and the influence of the source polarization is practically eliminated in the final results. PC2 was placed after the GILFP to compensate the polarization rotations caused by the single-mode fiber between the GILFP and the FFSC. To perform this task, FSP was oriented at 90° angle, then PC2 was finely adjusted to minimize the measured power at the FSPM. Afterward, FSP was rotated in 10° steps and the transmitted power was recorded for each step. The results of this test ate presented in Fig. 5a,b.

#### Investigation the effects of different coatings

The configuration in Fig. 4c was also used to examine the influence of each layer on the polarization and to validate the proposed theories. For this purpose, bare SPF, 30 nm As_2_S_3_ coated SPF, and PMMA/30 nm As_2_S_3_ coated SPF were placed instead of GILFP in the setup and the transmitted power at 1550 nm for different angles was measured and compared with that of the GILFP (Fig. 5c).

#### ER measurement

For almost all the tested wavelengths, the minimum transmitted power from the GILFP which was achieved by adjusting PC2 and the FSP was so low that it could not be measured by the FSPM. Also, the nominal ER of the FSP used in the setup was 50 dB. Therefore, higher ER values could not be measured by using this setup and another configuration was designed to measure the ER more accurately.

The more accurate ER measurement setup is shown in Fig. 4d. The polarization of the transmitted light from the source was parallelized with the transmission axis of an in-line fiber polarizer (ILP1) (Thorlabs, ILP1550SM-FC) by using PC1. PC2 does the same action for the second in-line fiber polarizer (ILP2). By adjusting these two PCs, the output power of ILP2 was maximized, ensuring the alignment of the output polarization of the source with the transmission axes of ILP1 and ILP2. This alignment results in a pure polarization state with a high ER which is ideally equal to the summation of the ER of the source and those of the two in-line polarizers. Afterward, PC3 was adjusted to minimize and maximize the transmitted power from the GILFP which was measured by a fiber coupled power meter (FCPM) (EXFO, FOT300) and the minimum and maximum values were recorded for ER calculation. This experiment was performed for different wavelengths with the same sources used in the previous test (Fig. 5d).

#### IL measurement

To measure the IL, a PC was placed between the source and the GILFP and the power was measured just after the GILFP and before any splices. By adjusting the PC, the transmitted power through the GILFP was maximized. When these conditions are met, the incident polarization to the GILFP is a linear polarization normal to the polished surface, i.e. TM polarization. This maximum power for various wavelengths was recorded as the transmitted TM power. Then, the fiber just before the GILFP and after all the splices was cleaved and the power for different wavelengths was measured. The ratio of the maximum transmitted power to the incident power is the IL of the device. In IL measurement test, FOT300 was used as the source for 1310 nm wavelength and mTLS-A1 was used for wavelengths in the range of 1520–1635 nm.

### Raman and SEM analysis

RAMAN measurements were conducted by utilizing a WiTec alpha300 RAS with excitation laser at 532 nm. To acquire Fig. 4b the PMMA/Graphene/As_2_S_3_ coated SPF was scanned with the Raman microscope. Then the FWHM of the 2D peak of the spectrum in each scan point was measured and mapped to the position of the point and a color scale graph was produced. If the FWHM is lower than 40 *cm*^−1^ the graphene is single layer which is the case for almost all the points in Fig. 4b^44^.

The SEM analysis was conducted by a JEOL JSM-7900F SEM microscope.

## Supplementary information

Supplementary information

Supplementary video

## Data Availability

The data that support the findings of this study are available on request from the corresponding author.
