# Supplementary material for: Utilizing polarization-selective mode shaping by chalcogenide thin film to enhance the performance of graphene-based integrated optical devices
Source: Sci Rep. 2019 Aug 27;9:12446. doi: 10.1038/s41598-019-48890-y (PMC6711980; doi:10.1038/s41598-019-48890-y)
Supplement: Supplementary file 1 — Supplementary information [file 41598_2019_48890_MOESM1_ESM.pdf]

Utilizing polarization-selective mode shaping by chalcogenide thin film to enhance the performance of graphene-based integrated optical devices

Hamed Nikbakht,† Hamid Latifi,*,†,‡ Gholam-Mohammad Parsanasab,¶ Majid Taghavi,† and Maryam Riyahi§

†*Laser and Plasma Research Institute, Shahid Beheshti University, Tehran 1983969411, Iran*

‡*Faculty of Physics, Shahid Beheshti University, Tehran 1983963113, Iran*

¶*Integrated Photonics Laboratory, Faculty of Electrical Engineering, Shahid Beheshti University, Tehran 1983963113, Iran*

§*Faculty of Science, Shahid Chamran University of Ahvaz, Ahvaz 6135743135, Iran*

E-mail: latifi@sbu.ac.ir

Supplementary Materials

1 Theory of Polarization Selective Mode Shaping

The difference in profiles of transverse electric (TE) and transverse magnetic (TM) modes, as will be described in the following, is a direct consequence of Maxwell's equations. A slab waveguide (Figure S1), as a two-dimensional (2D) counterpart of a side polished fiber (SPF)

coated with a high index layer, was analytically studied.

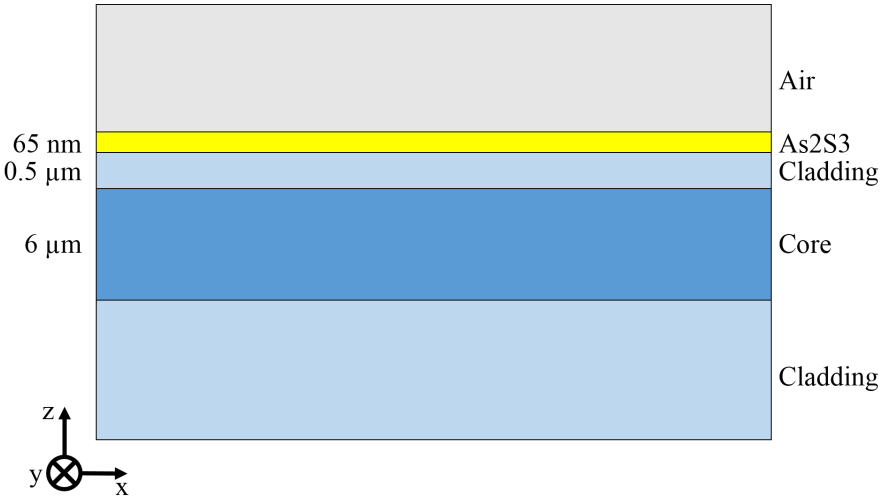

Figure S 1: Schematics of the 2D counterpart of a SPF with a high index overlaying layer.

For homogeneous and isotropic dielectric materials Maxwell's equations are as follows.

$$\begin{aligned} \nabla.\vec{E} &= 0 \\ \nabla.\vec{H} &= 0 \\ \nabla \times \vec{E} &= -\mu_0 \frac{\partial \vec{H}}{\partial t} \\ \nabla \times \vec{H} &= \epsilon_0 n^2 \frac{\partial \vec{E}}{\partial t} \end{aligned} \tag{1}$$

For a monochromatic light propagating in x direction we have

$$\begin{aligned} \vec{E} &= \vec{E}(y,z)e^{i(\omega t - \beta x)} \\ \vec{H} &= \vec{H}(y,z)e^{i(\omega t - \beta x)} \end{aligned} \tag{2}$$

Substituting equation 2 into equation 1 leads to the following equations:

$$\begin{aligned}
\frac{\partial E_z}{\partial y} - \frac{\partial E_y}{\partial z} &= -i\omega\mu_0 H_x \\
\frac{\partial E_x}{\partial z} + i\beta E_z &= -i\omega\mu_0 H_y \\
-i\beta E_y - \frac{\partial E_x}{\partial y} &= -i\omega\mu_0 H_z
\end{aligned}$$

$$\begin{aligned}
\frac{\partial H_z}{\partial y} - \frac{\partial H_y}{\partial z} &= i\omega\epsilon_0 n^2 E_x \\
\frac{\partial H_x}{\partial z} + i\beta H_z &= i\omega\epsilon_0 n^2 H_y \\
-i\beta H_y - \frac{\partial H_x}{\partial y} &= i\omega\epsilon_0 n^2 H_z
\end{aligned} \tag{3}$$

In the slab waveguide of Figure S1 the fields are independent of y coordinate. Then, two sets of independent equations are obtained.

$$\begin{aligned}
\frac{\partial E_y}{\partial z} &= i\omega\mu_0 H_x \\
i\beta E_y &= i\omega\mu_0 H_z \\
\frac{\partial H_x}{\partial z} + i\beta H_z &= i\omega\epsilon_0 n^2 E_y
\end{aligned} \tag{4}$$

$$\begin{aligned}
\frac{\partial H_y}{\partial z} &= -i\omega\epsilon_0 n^2 E_x \\
i\beta H_y &= -i\omega\epsilon_0 n^2 E_z \\
\frac{\partial E_x}{\partial z} + i\beta E_z &= -i\omega\mu_0 H_y
\end{aligned} \tag{5}$$

The first set describes TE modes and the latter describes TM modes. Therefore, TE mode must satisfy

$$\frac{\partial^2 E_y}{\partial z^2} + (k^2 n^2 - \beta^2) E_y = 0 \tag{6}$$

According to Maxwell's boundary conditions E_y and H_x components of the fields must be

continuous at the boundaries. Also, TM mode must satisfy

$$\frac{\partial^2 H_y}{\partial z^2} + (k^2 n^2 - \beta^2) H_y = 0 \tag{7}$$

where, H_y and E_x should be continuous at the boundaries.[1]

For TE mode E_y in layers 1-5 we will be

$$\begin{aligned}
E_1 &= b_1 e^{-\gamma_1 z} \\
E_2 &= a_2 \cos(K_{T2} z) + b_2 \sin(K_{T2} z) \\
E_3 &= a_3 e^{\gamma_3 z} + b_3 e^{-\gamma_3 z} \\
E_4 &= a_4 \cos(K_{T4} z) + b_4 \sin(K_{T4} z) \\
E_5 &= a_5 e^{\gamma_5 z}
\end{aligned} \tag{8}$$

Also for TM mode H_y in different layers will be

$$\begin{aligned}
H_1 &= b_1 e^{-\gamma_1 z} \\
H_2 &= a_2 \cos(K_{T2} z) + b_2 \sin(K_{T2} z) \\
H_3 &= a_3 e^{\gamma_3 z} + b_3 e^{-\gamma_3 z} \\
H_4 &= a_4 \cos(K_{T4} z) + b_4 \sin(K_{T4} z) \\
H_5 &= a_5 e^{\gamma_5 z}
\end{aligned} \tag{9}$$

where

$$\begin{aligned}
\gamma_1 &= \sqrt{\beta^2 - n_1^2 k^2} \\
K_{T2} &= \sqrt{n_2^2 k^2 - \beta^2} \\
\gamma_3 &= \sqrt{\beta^2 - n_3^2 k^2} \\
K_{T4} &= \sqrt{n_4^2 k^2 - \beta^2} \\
\gamma_5 &= \sqrt{\beta^2 - n_5^2 k^2}
\end{aligned} \tag{10}$$

By applying boundary conditions to these equations mode profiles in Figure S2 will be

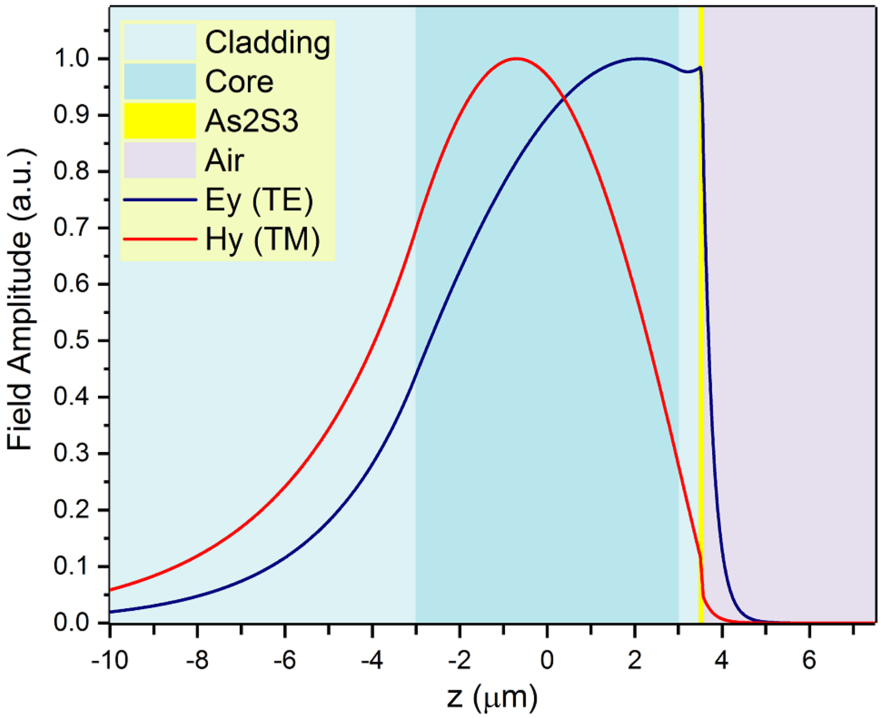

Figure S 2: TE and TM modes of a slab waveguide with a high refractive index coating.

achieved. In these calculations the refractive index (RI) of the core, cladding, and As_2S_3 are considered to be 1.4711, 1.4660, and 2.43, respectively. The dimensions of these layers are determined in Figure S1.

Figure S2 shows that formation of different mode profiles for different polarizations is a direct consequence of Maxwell's equations and by adjusting the thickness of layers, polarization selective mode shaping can be achieved.

2 Polarization Selective Mode Shaping with Different Coatings

Polarization selective mode shaping can be achieved with different high RI materials such as PMMA, Silicon, chalcogenide and so on. For example, Figure S3 shows mode profiles for side

polished fiber with different coatings. The thicknesses are approximately at the optimum value. It means that by increasing coatings thicknesses by a small amount (fewer than 5 nm for PMMA and fewer than 1 nm for As_2S_3 and Silicon) the structure becomes multi-mode. This simulation shows that PMMA alone is not strong enough to produce significantly

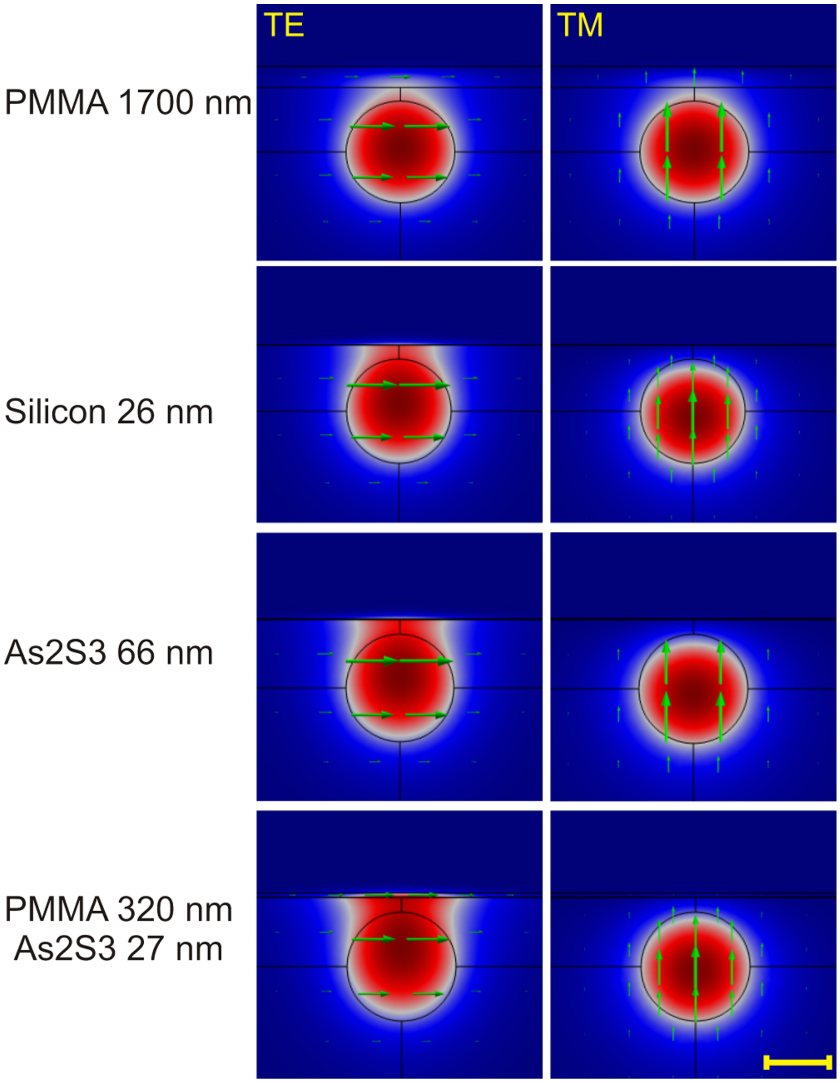

Figure S 3: TE and TM mode profiles for different coatings on the SPF, just before the structure becomes multi-mode.

different mode profiles. It also can be observe that, the interaction at the boundary is lower with silicon coating than chalcogenide. Since it is hard to control chalcogenide thickness in the optimum value, a combination of PMMA and chalcogenide layers is used to fabricate the polarizer.

3 Liquid Drop Test

The polished depth is required to be determined for the optimization of the coating thicknesses. This parameter was approximated by using the liquid drop experiment. In this experiment, different liquids with refractive indices were poured around the bare SPF and the corresponding transmitted powers were measured. To calculate the polished depth, these data were fitted to the expected transmitted power ($e^{-\alpha l_p}$). Where, l_p is the polished length of the fiber and α is the extinction coefficient, which can be calculated by Leminger and Zengerle formula,[2,3]

$$\alpha = \frac{4\beta}{n_{cl}^2 k_0^2}\left[\frac{u}{aVK_1^2(w)}\right]^2 \frac{V_{ex}^2 - w^2}{V_{ex}^2}\int_0^1 \sqrt{1-x^2} exp\left(\frac{-2d}{a}\sqrt{\left(V_{ex}^2 - w^2\right)x^2 + w^2}\right) dx \quad (11)$$

which describes the attenuation of the light in SPFs. In this formula, n_{cl}, k_0, β_0, a, V, w, u and d, are cladding refractive index, propagation constant in vacuum, propagation constant in the un-polished fiber, core radius, V number, transverse propagation constants in the core and cladding and polished depth, respectively. V_{ex} is the modified V number, defined as $V_{ex} = \frac{2\pi a}{\lambda}\sqrt{n_{ex}^2 - n_{cl}^2}$, in which n_{ex} is the refractive index of the liquid which is around the SPF.

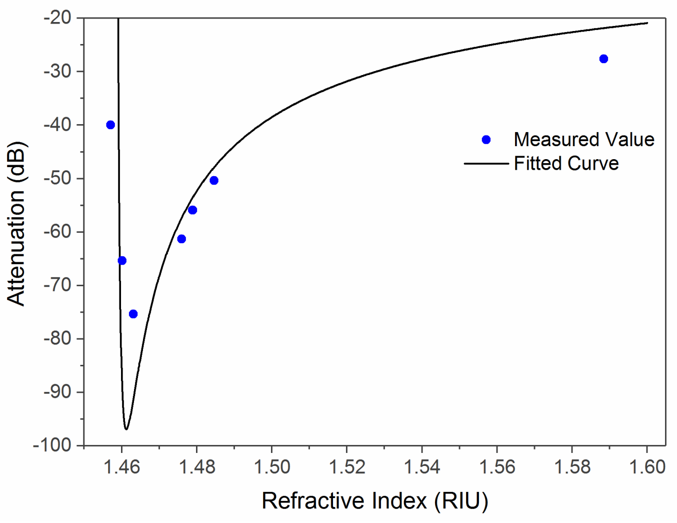

Figure S 4: Liquid drop experiment results fitted to the theoretical curve. (Refractive indices were acquired from refs[4–6])

The fitting determines the polished depth to be about $5.2 \pm 0.2\ \mu m$. However, the quality of fitting was not satisfying, and this value was not determined accurately. Therefore, care should be taken in choosing coating thicknesses so that this error can be compensated later in the experiment. The using of PMMA overlay enables the thickness adjustment of this layer throughout the experiment. To adjust the thickness of PMMA layer accurately, the extinction ratio (ER) of the fabricated GILFP was monitored and it was used as a feedback for determining optimum PMMA thickness. To increase this thickness by one step, the GILFP was spin coated by 2% PMMA in anisole solution at the speed of 4000 rpm. To reduce the PMMA thickness, the GILFP was fixed with the slope of 30° and, a drop of anisole was dropped on it. After each step, the GILFP was left for one hour for the anisole to be vaporized, then the ER was measured. By following this procedure, the optimum PMMA thickness was achieved.

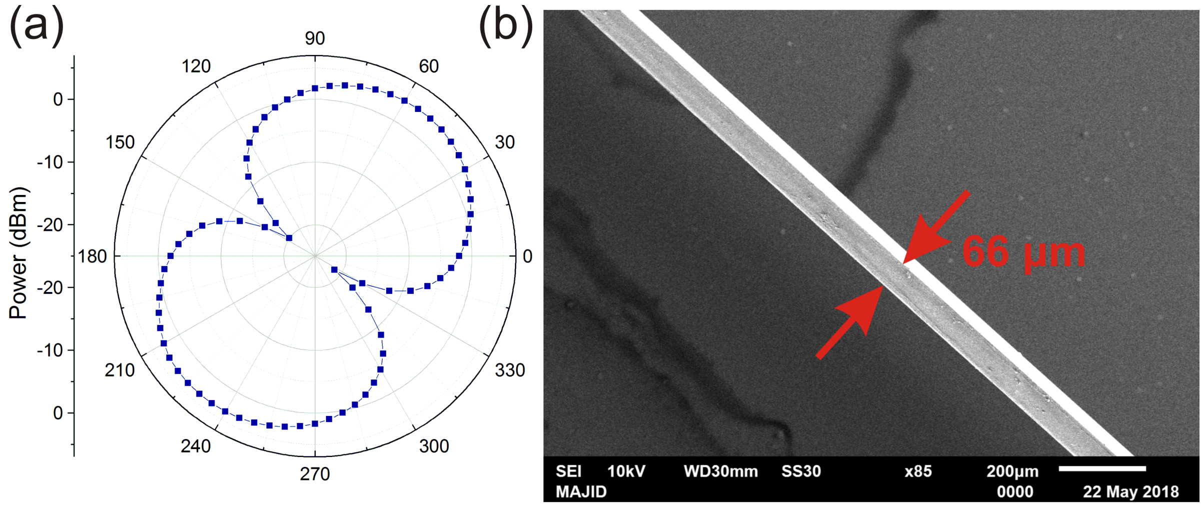

Figure S 5: (a) Transmitted power at different FSP orientations for when the fiber is cleaved 2 cm away from the polished section. (Angle is measured relative to the microscope slide surface), (b) SEM image of the GILFP after cleaving which was used to determine the fiber orientation relative to the microscope slide.

4 TM-Pass Confirmation Test

To determine which polarization passes through the polarizer, the fiber was detached from the slide and then it was cleaved from a point 2 cm away from the polished section of the GILFP. Then, it was fixed again to the microscope slide. The transmitted power from the cleaved end was parallelized by a lens and passed through a free-space polarizer (FSP) and the transmitted powers at different FSP orientations were measured by a free-space power meter (FSPM). Due to the short length of the fiber after the polished section, the polarization rotation in the fiber is virtually eliminated, and it is expected to measure the maximum transmitted power when the transmission if the FSP is perpendicular to the polished surface of the GILFP (TM polarization). The results of this test is presented in Figure 5a, and the angles in this graph is the angle of the FSP transmission axis relative to the microscope slide surface. The maximum power is observed at 55°.

In the cleaving procedure the fiber was rotated relative to the slide, and its polished surface was no longer parallel with the slide. To determine the angle between these two surfaces, SEM image of the GILFP without any tilt was acquired (Figure 5b). The image confirms the rotation of the fiber relative to the slide. The width of the polished surface in this image was measured through the SEM image to be 66 μm. This value is the projected width of polished surface to a plane parallel to the slide surface. Considering the polished depth of 5.2 μm the real polished surface width would be 124.5 μm. Therefore, the fiber's rotation angle is calculated to be $\theta = ArcCos(66/124.5) = 58\,\text{deg}$, which is approximately equal to the value obtained for the angle at which the maximum power is transmitted.

References

(1) Okamoto, K. *Fundamentals of Optical Waveguides*; 2006.

(2) Leminger, O.; Zengerle, R. Determination of single-mode fiber coupler design parameters from loss measurements. *J. Lightwave Technol.* **1985**, *3*, 864–867.

(3) Tseng, S.-M.; Chen, C.-L. Side-polished fibers. *Appl. Opt.* **1992**, *31*, 3438–3447.

(4) Saunders, J. E.; Sanders, C.; Chen, H.; Loock, H.-P. Refractive indices of common solvents and solutions at 1550 nm. *Appl. Opt.* **2016**, *55*, 947–953.

(5) Kedenburg, S.; Vieweg, M.; Gissibl, T.; Giessen, H. Linear refractive index and absorption measurements of nonlinear optical liquids in the visible and near-infrared spectral region. *Opt. Mater. Express* **2012**, *2*, 1588–1611.

(6) Rheims, J.; Köser, J.; Wriedt, T. Refractive-index measurements in the near-IR using an Abbe refractometer. *Meas. Sci. Technol.* **1997**, *8*, 601–605.
